# Early Hip Fracture Surgery in Patients Taking Direct Oral Anticoagulants Improves Outcome

**DOI:** 10.3390/jcm13164707

**Published:** 2024-08-11

**Authors:** Benjamin K. Devlieger, Pol M. Rommens, Andreas Baranowski, Daniel Wagner

**Affiliations:** 1 Department of Orthopaedics and Traumatology Westpfalz-Klinikum, 67655 Kaiserslautern, Germany; 2 Department of Orthopaedics and Traumatology, University Medical Center of the Johannes Gutenberg University, 55131 Mainz, Germany; 3 Klinikum Anbach, 91522 Ansbach, Germany; 4 Department of Orthopaedics and Traumatology, University Hospital of Lausanne, CH-1011 Lausanne, Switzerland

**Keywords:** proximal femur, anticoagulant, geriatric, osteoporotic fracture, complications

## Abstract

**Background/Objectives**: The increasing numbers of already endemic hip fractures in the elderly taking anticoagulants is a growing concern for daily surgical practice. Ample evidence demonstrates decreased morbidity and mortality in the general population when surgery is performed at the earliest possibility. Direct anticoagulants are relatively new drugs that can cause increased perioperative bleeding. Current guidelines propose stopping the drug to allow for elimination before performing elective surgery. Optimal management in urgent hip surgery is presently based on expert opinion with arbitrary cut-offs. In this study, we investigated whether patients taking direct anticoagulants would benefit from early surgical treatment, regardless of the timing since last intake. **Methods**: A total of 340 patients were included in the analysis, of which 59 took direct anticoagulants. The primary outcomes were time to surgery, postoperative transfusion rate, postoperative hemoglobin decrease, length of postoperative in-hospital stay (LOPS), revision rate, and complication rate (medical and surgical). **Results:** Our findings showed that the anticoagulated group was fit for discharge earlier when operated on within 24 h (*p* = 0.0167). Postoperative transfusion and medical complication rate tended to be lower when the operation was performed earlier. Revision rate due to hematomas were higher in the direct anticoagulant group without a relationship to time to surgery. Simple linear regression could not determine a relationship between postoperative hemoglobin change and time to surgery. **Conclusions:** We suggest that directly anticoagulated patients needing hip fracture surgery must be considered for early surgery.

## 1. Introduction

With a continuously ageing population in Germany and Europe, the occurrence of proximal femoral fractures (PFFs) in the elderly is rapidly increasing. Concurrent rise of cerebrovascular and cardiovascular disease implies that a significant proportion of patients with proximal femur fractures also take anticoagulants. A new generation of direct anticoagulants is a growing class and has been approved for multitude of indications [1]. Dabigatran, Apixaban, Rivaroxaban, and Edoxaban were introduced from 2008 on, and the incidence of their use has since grown to 4–10% of PFF patients [2,3]. Although these drugs provide ease of use and safety while alleviating chronic risk of stroke and thrombosis, their effect on perioperative morbidity and mortality in emergent hip surgery is not fully elucidated. Accordingly, counteracting the anticoagulation effect is generally desired to reduce possible bleeding complications. Specific reversal agents exist for Dabigatran, Apixaban, and Rivaroxaban; however, these are still expensive, scarcely available, and often reserved for major trauma with active bleeding. Alternatively, waiting for effect reversal through renal and hepatic elimination usually takes 24 to 48 h depending on renal status and specific drug. In elective surgery, guidelines suggest pausing DOACs 24–96 h before conducting anesthesia [4]. In contrast to this, proximal femur fractures essentially require early surgical treatment (<24 h), as it supports optimal postoperative outcomes and reduces one-year mortality and morbidity [5,6]. We therefore sought to examine if patients taking DOACs differ in time to surgery or had more complications, if there was a different outcome in patients operated on in <24 h or >24 h, and if there was a higher risk if patients with DOACS are operated on within 24 h. We hypothesized that the positive effect of early operative treatment outweighed the negative effect of perioperative bleeding complications in patients taking DOACs. 

## 2. Materials and Methods

We conducted a retrospective observational study. The study protocol was approved by the local ethical committee, where the requirement to obtain informed consent was waived (ethics commission of the state chamber of medicine of Rhineland-Palatinate, Ref 837.140.17 (10,974)). Patient hospital records from a single academic hospital were searched for admissions between 1 January 2018 and 31 December 2019 for patients with a PFF using the following ICD-10 codes: S72.0, S72.1, and S72.2. Inclusion criteria were operative management of a PFF within 48 h after admission. Patients with a TTS > 48 h were not included, as this was often the result of a medical issue that had to be treated first. Other exclusion criteria were revision surgery, periprosthetic fractures, polytrauma, pathological fracture and multistage operative therapy, preoperative intensive care treatment, preoperative blood transfusions, and documentation errors. A total of 340 patients were included for analysis after processing a total of 462 records. Using a data extraction template, relevant data on each individual patient were retrieved. Outcomes were defined as time to surgery (TTS), postoperative transfusion rate, postoperative hemoglobin decrease, length of postoperative in-hospital stay (LOPS), revision rate after at least one year, and complication rate. Moreover, the number and type of surgical and medical complications was recorded rather than the number of patients who suffered a complication. 

A secondary analysis comparing the patients operated on between 0 and 24 h and 24 and 48 h was planned, with the same primary outcomes except TTS. In our center, the transfusion trigger was <7 g/dL for all patients and <8 g/dL when patients where symptomatic or had cardiovascular comorbidities. Postoperative anticoagulation was managed with a therapeutic dose of low-molecular-weight heparin for at least one week, until the wounds were dry and showed no signs of infection, after which it was switched back to the patient’s previous anticoagulation.

Patient characteristics and outcomes were identified by reading the admission notes, the anesthesia reports, and the physician’s discharge letter. The data were extracted following a premade template including several clinical and laboratory presentations other than the above defined primary outcomes. Pharmacokinetic differences between DOACs and their influence on the outcomes could not be evaluated due to lack of routine laboratory plasma concentration evaluation in our center at the time. Data were collected in Excel (Microsoft Corporation, Redmond, WA, USA) and prepared for statistical purposes. Statistical analysis was performed through Prism GraphPad (GraphPad Prism version 8.0.0 for Windows, GraphPad Software, San Diego, CA, USA). Binary data were examined using Fisher’s exact test for two and chi-squares test when dealing with multiple variables. Continuous data were judged on normality using histograms and QQ plots, as well as statistical normality testing (Anderson–Darling test, D’Agostino and Pearson test, Shapiro–Wilk test, and Kolmogorov–Smirnov test). Normality was assumed when at least three tests indicated normality. In examining hemoglobin decrease, there were a total of six missing values, and one outlier was removed through ROUT testing with Q = 1%. When dealing with normally distributed data, an unpaired *t*-test was used; for nonparametric data, a Mann–Whitney test was used. In the case of multiple variables, a one-way ANOVA for normally distributed and Kruskal–Wallis test for nonparametric distributed data were used. Simple regression analysis was used to determine if a correlation existed between time to surgery and hemoglobin change as well as between time to surgery and length of postoperative in-hospital stay. A power analysis determined that the patient group was not large enough to perform multivariate regression. Statistical significance was set as *p* < 0.05 throughout. An authority provided approval and the corresponding ethical approval code.

## 3. Results

Of the 340 patients included, 59 patients were taking DOACS (17%), 26 were taking coumarins (8%), and 255 patients were taking no anticoagulants (75%). Of the patients taking DOACS, 33 were taking Apixaban (55%), 18 Rivaroxaban (31%), 5 Dabigatran (8%), and 3 Edoxaban (5%). Antiaggregant status in each group as well as other demographics are summarized in Table 1. Mean age was comparable for all groups. The DOAC group was slightly more female-dominant and had a higher average ASA score. Most patients (225, 66%) were operated on during business hours. The population included 187 osteosyntheses and 153 hemiprothesis. A total of 140 patients (41%) developed an in-hospital complication, most of which were medically related. A total of 22 patients (6.5%) suffered a total of 27 surgically related complications (Table 2).

**Figure 1:**
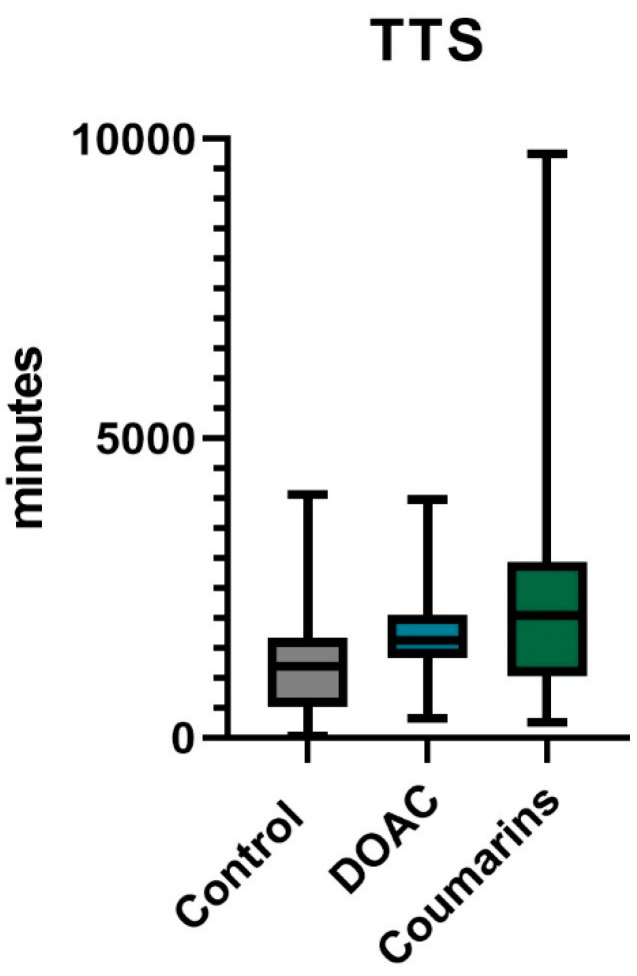
The comparison of time to surgery (TTS, in minutes) in patients taking no anticoagulants (grey), taking direct anticoagulants (blue), and in patients taking coumarins (green).

In our patient group, the TTS for patients taking coumarins was longer than for patients taking DOACS, which, in turn, was longer than the control group (Figure 1, *p* < 0.0001). A total of 36% of patients taking any form of antithrombotics (antiaggregant or anticoagulant; *n* = 192) needed a postoperative transfusion, which was similar to patients not taking any antithrombotics (34%; *n* = 110). Similarly, no significant difference in transfusion rate was noted when comparing the DOACs to the control group (*n* = 59, *n* = 255 respectively; *p* = 0.8759). Three main analyses were performed to examine the primary outcomes.

**Figure 2:**
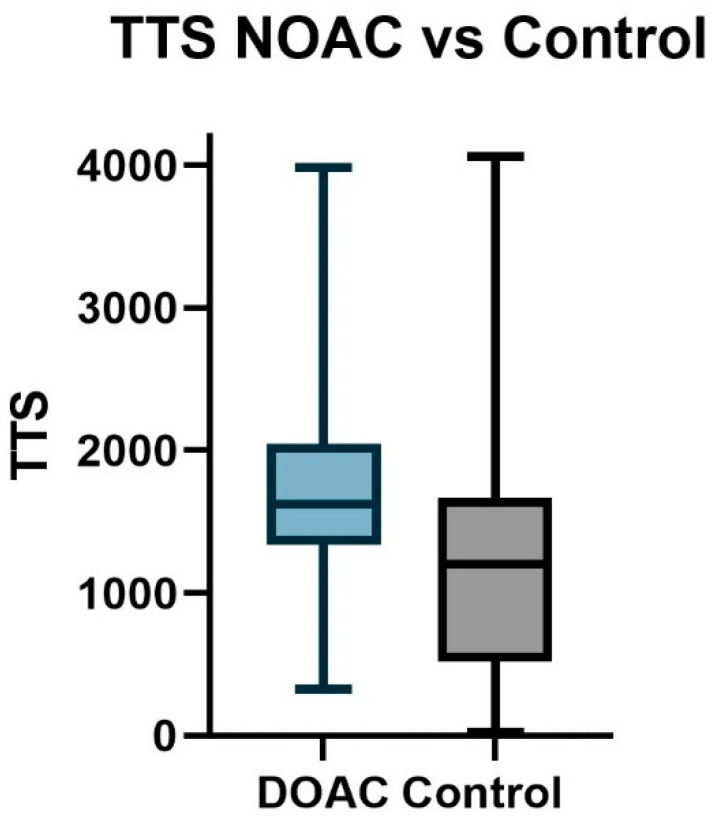
Magnification of Figure 1, showing a clear difference in operative delay between patients taking no anticoagulants (grey) and patients taking direct anticoagulants (blue).

The first analysis compared patients taking DOACs with a control population taking no anticoagulants. Patients taking DOACS had a significantly longer time to surgery than the anticoagulant-free control group (Figure 2, *p* < 0.0001). No significant differences could be distinguished in the other outcome measures (Table 3 (1)).

**Figure 3:**
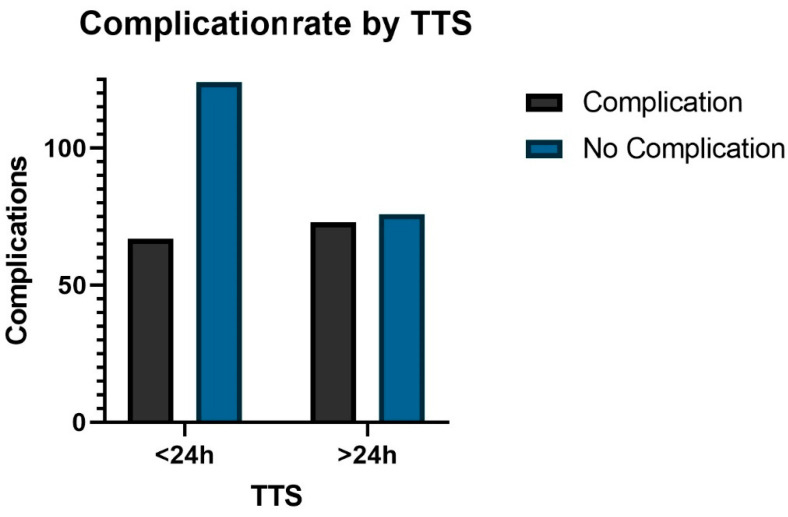
The complication rate related to time to surgery (TTS, minutes).

The second analysis examined the difference in outcome of patients who were operated on within 24 h and of patients operated on between 24 and 48 h. Patients who were operated on within 24 h were less likely to need a transfusion (*p* = 0.0138), had fewer complications (*p* = 0.0108, surgical and medical), and were less likely to be taking a DOAC (Figure 3, *p* < 0.0001). Length of postoperative stay seemed to be shorter, with similar postoperative change in hemoglobin (*p* > 0.05; Table 3 (2)).

**Figure 4:**
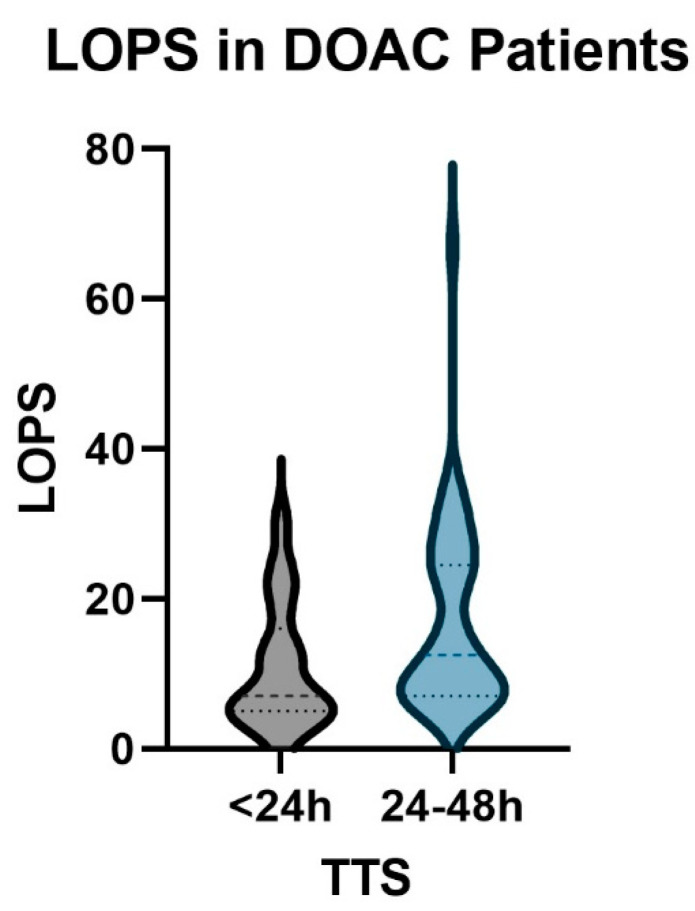
The relationship between the length of postoperative stay (LOPS, days) and time to surgery (TTS, minutes). Patients operated on within 24 h after admission (grey) are discharged earlier than those operated on between 24 and 48 h (blue).

A comparison of DOAC patients operated on within 24 h to those operated on between 24 and 48 h was conducted to determine if the results of previous analyses were expandable to the DOAC population. Patients operated on in <24 h had lower transfusion rates with similar hemoglobin change and had fewer complications and revisions; however, all were without statistical significance (Table 3 (3)). The length of postoperative stay was 6 days less for the group operated on earlier (Figure 4, *p* = 0.0167).

**Figure 5:**
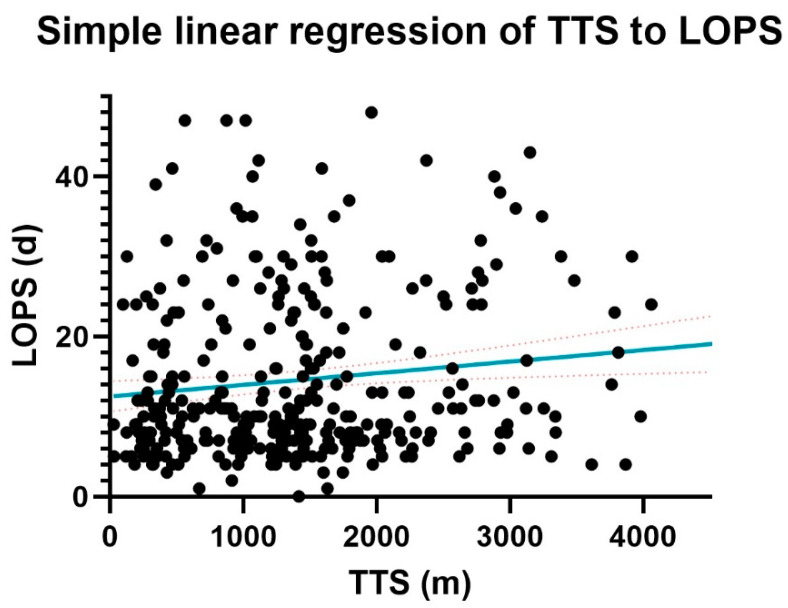
Simple linear regression of time to surgery (TTS) and length of postoperative stay (LOPS).

Outcomes of simple linear regression could not determine a relationship between postoperative hemoglobin change and time to surgery (*p* = 0.9398). Time to surgery (minutes) and length of postoperative stay (days) were correlated in the whole patient population (Figure 5, R2 = 0.02025; *p* = 0.0086, slope 0.001450). Separate analyses could not prove any such correlation in the DOAC subpopulation (*p* = 0.1033, R2 = 0.04587) or the control subpopulation (*p* = 0.2310, R2 = 0.005665).

**Figure 6:**
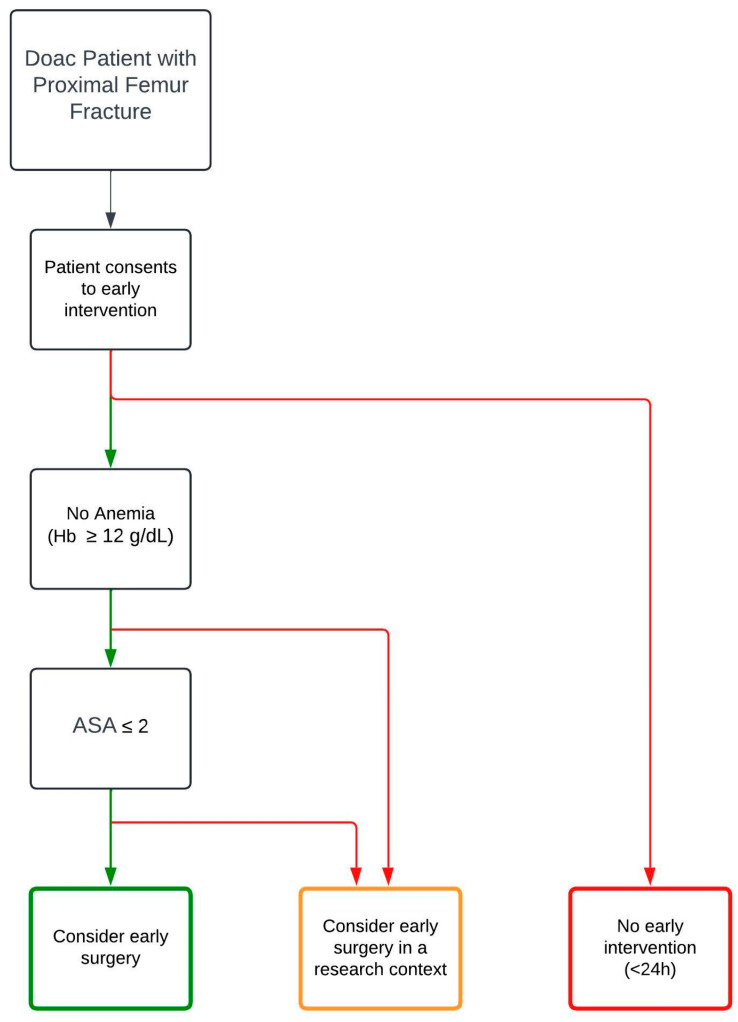
Potential pathway for DOAC patients with a proximal femur fracture. Green arrows signify a positive answer, and red a negative.

After two years, 26 patients needed revision surgery: 16 during the same hospital stay, 14 in the control population (2 hematoma, 8 infections, 3 other revisions), 7 in the DOAC population (4 hematoma, 3 infections), and 5 in the coumarins group (2 hematoma, 3 infections). Eight implant removals in the control group were not included in the analysis because we did not consider this to be a complication. A significant difference in revision rate between the groups could not be established. When only regarding hematomas, the DOAC subgroup had significantly more hematomas when compared to the control group (*p* = 0.0127). Of the four hematomas needing revision in the DOAC group, all patients had normal kidney function. The prescribed DOAC was Rivaroxaban in two patients and Apixaban in the two others. Three of four patients had been operated on after 24 h (Table 4). A potential pathway to treat these patients is presented in Figure 6.

## 4. Discussion

Patients taking DOACs are a growing subgroup of patients presenting with PFFs. The longer time to surgery for these patients has been well documented [7,8,9,10] and is most likely influenced by cautious guidelines on perioperative management of DOACs in elective surgery [4,11]. Choosing a safe timeframe for emergent surgery is crucial and should ideally be based on sound data analysis specific to this patient subgroup. Due to the longer mean waiting time to surgery for DOAC patients in our patient collective, comparisons between anticoagulated patients and non-anticoagulated controls could only be accurately made within a 48 h timeframe to ensure accuracy. Nonetheless, the DOAC subgroup analyses in this study support early surgical management of patients with a proximal hip fracture, regardless of their DOAC status.

The literature to date has reported mixed data on transfusion need in DOAC patients, with no clear relationship to time to surgery [12,13]. In our overall sample, blood transfusions were needed more often when time to surgery increased. This effect was highlighted in DOAC patients, where 38% needed a transfusion when operated after 24 h compared to 18% of those operated on earlier. This suggests a similar perioperative blood loss between DOAC patients and controls and provides insight that other factors may be influencing transfusion rate more than DOAC status. This is an effect that has been previously reported on, most recently by Levack et al. in 132 patients taking DOACs [14]. Similarly, perioperative hemoglobin change was relatively stable between 2.4 and 2.7 g/dL throughout all performed analyses. Another report by Kolodychuk et al. showed that the postoperative hemoglobin drop was 0.8 g/dL higher in non-anticoagulated controls, compared to a DOAC group [15]. Growing evidence of similar hemoglobin change, irrespective of DOAC status or time to surgery, undermines the rationale for delaying surgery for DOAC patients [7,8,16,17].

Furthermore, studies on perioperative blood loss and time to surgery have emphasized that early surgery reduced the need for transfusion in patients taking DOAC medication [14,18]. This is a crucial consideration, as perioperative blood loss in hip fractures can be up to six times higher than intraoperatively estimated blood loss [19]. More factors must be taken into consideration instead of only anticoagulation status, as past studies have shown that BMI, time to surgery, and fracture type have more impact on blood loss in proximal femur fracture than anticoagulation status [20]. Our study could not establish a significant link between surgical delay and hemoglobin change, in line with prior research [21].

Length of postoperative stay was not significantly longer in DOAC patients compared to controls. In contrast, a clear benefit of six days was seen in DOAC patients who received operative care within 24 h (*p* = 0.0167), an indication that the benefits of early operative management in geriatric hip fractures are still present in patients taking DOACs. A previous study of 28 DOAC patients shows supportive data, showing that a delay longer than 48 h resulted in a mean lengthening of seven days [22].

A positive linear regression correlation between time to surgery and postoperative length of stay was established in the entire patient population, though its effect could not be repeated on the isolated DOAC group. This is most likely due to an insufficient sample size, as the effect was closer to statistical significance with a steeper slope than the regression analysis in the control subgroup, despite larger size (*p* = 0.10 for *n* = 59 vs. *p* = 0.23 for *n* = 255).

Our DOAC patients had a similar rate of combined medical and surgical complications compared to controls. Treatment before 24 h was beneficial for all patients, reducing the complication rate by 14% (*p* = 0.0167). A similar effect was seen in the DOAC subgroup, where earlier surgery resulted in a 11% decrease in complication rate (*p* > 0.05). To our knowledge, data on medical complications after early operative management in DOAC patients have not previously been reported on. This provides better insight into the true resources that are needed to treat these patients.

The two-year revision rate was similar between DOAC patients and controls, although DOAC patients had significantly more revisions for hematomas. When looking at DOAC patients separately, a longer time to surgery does not decrease revision rate, and an overall decrease in revisions is seen when operated on withing 24 h. A total of 75% of hematomas needing revision were initially operated on more than 24 h after admission and, thus, were not prevented by waiting longer than 24 h. This suggests that waiting is not the treatment of choice to avoid revision surgery. Other authors have previously reported on 30-day revision rate. Franklin et al. had no revisions in the DOAC subgroup after 30 days, while Mullins et al. reported a 5% revision [7,21]. The difference in revision rate in our series can be partially explained by the inclusion of elective implant removals and the longer follow-up period of two years. When these are excluded, the overall two-year revision rate is 7.6%. When only considering hematoma and infection, the revision rate is 5.5%, respectively.

The authors contend that the current practice of waiting for passive reversal of DOAC activity lacks supports by prospective data and should therefore not be the default used when determining optimal surgical timing for patients taking DOACs. Traditionally cited large-scale studies for mortality in trauma patients taking anticoagulants were often conducted before DOAC drug approvals in 2008 or do not differentiate between coumarin and DOAC patients. Consequently, the perceived bleeding risk in this patient group might be overestimated due to the insufficient evidence to the contrary. Notably, a recent study by Bläsius et al., using the German Trauma Registry^®^, found higher mortality among patients on coumarins, but not among those taking DOACs, highlighting the need for specific data in this context [23].

Furthermore, displaced fractures can cause extensive bleeding without timely intervention. Because early surgical treatment of PFFs so clearly benefits patients without anticoagulation, the approach to early surgery in DOACs patients with PFF should be investigated thoroughly. Emerging research on this early operative window increasingly supports the safety of early hip surgery in patients taking DOACs, advocating against unnecessary delays [7,9,21]. In the absence of orthopedic trauma guidelines for DOAC patients with a hip fracture, waiting for the coagulative effect to wear off, as is preferred in elective surgery, could increase the well-documented risk of mortality in this frail population, while feared bleeding complications remain theoretical. These effects have been thoroughly investigated and proven to such an extent that many countries have active legislation to discourage unnecessary delays in treatment with “best tariff” reimbursement, fines, and legal procedures.

The strength of the study is its novel collection of data on perioperative medical complications among this patient group alongside the two-year revision rate in this patient population. A source of bias could involve the fact that generally healthier patients would be approved for surgery faster, regardless of DOAC intake, positively skewing the results of the examined earlier treatment subgroups. Further studies with a prospective design will be needed to translate the current findings into clinical guidelines

## 5. Conclusions

The results of this study suggest that waiting for urgent hip surgery in DOAC patients is unnecessary. Patients on direct oral anticoagulation waited significantly longer for surgery than their peers not taking any anticoagulation. The concept of early surgical care benefits hip fracture patients, despite DOAC intake. This study, therefore, supports previous reports that it might be safe to operate early in this specific patient population.

## Figures and Tables

**Table 1:** Patient characteristics (*n* = 340).

	Total (*n* = 340)		Coumarins (*n* = 26)		DOAC (*n* = 59)		No Anticoagulation (*n* = 255)	
Demographics	Mean	SD	Mean	SD	Mean	SD	Mean	SD
Age	80	12	85	8	84	8	79	12
Weight (kg)	69	17	71	12	68	14	68	18
Height (cm)	167	10	168	10	168	8	167	9.7
Male/Female	116/224 (34/66%)		9/17 (35/65%)		18/41 (31/69%)		89/166 (35/65%)	
Trauma	#	%	#	%	#	%	#	%
Fall from standing	296	87%	23	88%	51	86%	222	87%
High energy	4	1%	0	0%	0	0%	4	2%
Other low energy	14	4%	1	4%	4	7%	9	4%
Fall from bicycle	15	4%	0	0%	3	5%	12	5%
Fall from stairs	9	3%	2	8%	0	0%	7	3%
Unspecified	2	0%	0	0%	1	2%	1	0%
Operation	#	%	#	%	#	%	#	%
Osteosynthesis	187	55%	14	54%	29	49%	145	57
Hemiarthroplasty	153	45%	12	46%	30	51%	110	43
Timing of the operation								
Daytime (08–16 h)	225	66%	18	69%	46	78%	161	63%
Evening (16–00 h)	84	25%	6	23%	10	17%	68	26%
Night (00–08 h)	31	9%	2	8%	3	5%	26	10%
	Mean	SD	Mean	SD	Mean	SD	Mean	SD
Operating Time (min)	66		62		67		66	
Comorbidities			Mean	SD		SD		SD
ASA Score	2.8		3.3	0.55	3.1	0.87	2.7	0.97
Type of Comorbidity:	#	%	#	%	#	%	#	%
Cardiovascular	304	89%	26	100%	59	100%	219	86%
Diabetes	77	23%	3	12%	11	19%	63	25%
Pulmonary	84	25%	7	27%	12	20%	65	25%
Neurological	196	58%	14	53%	39	66%	143	56%
Rheumatological	18	5%	1	4%	2	3%	15	6%
History of cancer	69	20%	5	19%	8	14%	56	22%
Medication								
Antithrombotics	116	34%	1	3%	8	14%	107	41%
Admission Labs	Mean		Mean	SD	Mean	SD	Mean	SD
Creatine	1.1		1.3	0.5	1.0	0.3	1.1	0.5
C-Reactive Protein	18		16	32	17	27	18	34
Quick	87		36	22	65	23	97	16
INR	1.3		2.4	0.9	1.4	0.5	1.1	1.7
ApTT	29		35	6.3	30	7.1	28	11
Fibrinogen	348		334	110	327	92	354	111
White Blood cell Count	10.6		12.0	6.3	9.9	3.9	10.6	3.9
Hemoglobin	12.5		12.4	2.0	12.4	1.7	12.5	1.7
Thrombocyte count	243		216	85	236	95	247	79

#: Number.

**Table 2:** Complications (*n* = 232 in 140 patients).

Surgery related:	**27**
Implant malpositioning	3
Secondary dislocation of bipolar femoral head and fracture displacement	4
Intraoperative bleeding	1
Intraoperative periprosthetic fracture	4
Postoperative hematoma	2
Surgical wound infection	9
Persistent wound drainage	3
Nerve palsy	
- Sensory	1
- Motor	0
Medically related:	**205**
Pneumonia, lung disease exacerbation	39
Cardiovascular events, CV disease exacerbation	42
Deep venous thrombosis	1
Pulmonary embolism	1
Urinary tract infection	33
Acute kidney failure	24
Liver/gallbladder/pancreas/ileus dysregulation	7
Pressure sores	6
Delirium	21
GI bleeding	8
Neurological event, neurological disease exacerbation	8
Sepsis	3
Other (bursitis, dehydration, diabetic complications, transfusion reaction, acute limb ischemia, severe postoperative pain)	12

**Table 3:** (1) Outcomes after hip fracture surgery in patients taking DOACs (*n* = 59) compared to no anticoagulation (*n* = 255). Variables are expressed as the mean and standard deviation unless stated otherwise. (2) Outcomes after hip fracture surgery in all patients operated on within 24 h (*n* = 191) compared to 24–48 h (*n* = 149), expressed as the mean and standard deviation unless stated otherwise. (3) Outcomes after hip fracture surgery in patients taking DOACs operated on within 24 h (*n* = 17) compared to 24–48 h (*n* = 42), expressed as the mean and standard deviation unless stated otherwise.

(1)			
	DOAC	No Anticoagulation	*p*
Transfusion rate (%)	30.6%	32.2%	0.8759
Hemoglobin change (g/dL)	2.4 + 1.3	2.7 +1.4	0.1698
Length of postoperative stay (days)	15.1 + 11.7	14.4 + 10.2	0.9838
Time to surgery (hours)	28.63 + 12.28	21.35 + 14.93	**<0.0001**
Patients with complications (%) - Of which surgical Surgical complications (#) Medical complications (#)	37.3% 4 (6.8%) 6 (10%) 33 (56%)	41.2% 14 (5.5%) 18 (7%) 154 (60%)	0.6596
Revisions total (%) Revision same stay - Hematoma - Infection/wound complication - Pseudarthrosis, AVN	7 (11.8%) 2 (3.4%) 4 3 0	13 (5.1%) 11 (4.3%) 2 8 3	0.0726
(2)			
	<24 h	24–48 h	*p*
Transfusion rate (%)	50 (25.9%)	58 (39.45%)	**0.0138**
Hemoglobin change (g/dL)	2.69 + 1.37	2.630 + 1.364	0.7144
Length of postoperative stay (days)	13.7 + 10.0	15.8 + 11.1	0.0711
Complications general (%)	35.1%	49.0%	**0.0108**
Complications in DOAC patients (%)	9.3%	28.8%	**<0.0001**
(3)			
	<24 h	24–48 h	*p*
Transfusion rate (%)	17.7%	38.1%	0.2179
Hemoglobin change (g/dL)	2.58 + 1.1	2.39 + 1.4	0.6077
Length of postoperative stay (days)	10.8 + 8.3	16.8 + 12.4	**0.0167**
Complications (%)	29.4%	40.5%	0.5561
Revisions (%)	5.9%	11.9%	0.6622

#: Number.

**Table 4:** Revisions.

	Control	DOAC	AVK
Hematoma	2 (0.7%)	4 (6.7%)	2 (7.7%)
Infection and wound healing problems	8	3	3
Pseudarthrosis, avascular necrosis	3	0	0
Total	13	7	5

## Data Availability

The deidentified datasets used and/or analyzed during the current study are available from the corresponding author on reasonable request.
